# Introduction: Special Issue on Vitamin D Dedicated to the Memory of Anthony W Norman

**DOI:** 10.1002/jbm4.10445

**Published:** 2020-12-21

**Authors:** Roger Bouillon, Mark Haussler, Dan Bikle, Sylvia Christakos, JoEllen Welsh

**Affiliations:** ^1^ KU Leuven Laboratory of Clinical and Experimental Endocrinology, Department of Chronic Diseases, Metabolism and Ageing Herestraat Leuven VB 3000 Belgium; ^2^ University of Arizona College of Medicine–Phoenix, Ringgold Standard Institution–Basic Medical Sciences Phoenix AZ USA; ^3^ University of California San Francisco Ringgold Standard Institution–Medicine San Francisco CA USA; ^4^ Department of Microbiology, Biochemistry and Molecular Genetics Rutgers University Newark NJ USA; ^5^ State University of New York at Albany, Ringgold Standard Institution–Environmental Health Sciences Albany NY USA

Anthony (Tony) W Norman was born into an “academic” family in 1938 in Ames, Iowa. He obtained his masters and doctoral degrees at the University of Wisconsin–Madison. His PhD thesis dealt with “the preparation, distribution and metabolism of tritiated vitamin D" (1963) with Hector F DeLuca as mentor.^1^ He thereafter moved to California for a postdoctoral fellowship at UCLA in the laboratory of Nobel laureate Paul D Boyer, followed by a first appointment at the University of California–Riverside. There, he was promoted over the years from assistant to full/distinguished professor and chairman of the department of biochemistry. His scientific career was fully devoted to virtually all aspects of vitamin D. Tony Norman was not only a very original hard‐working scientist; he was also a very successful mentor. Two recently published “In Memoriams” provide further details about his career.^2^, ^3^

**Fig 1:**
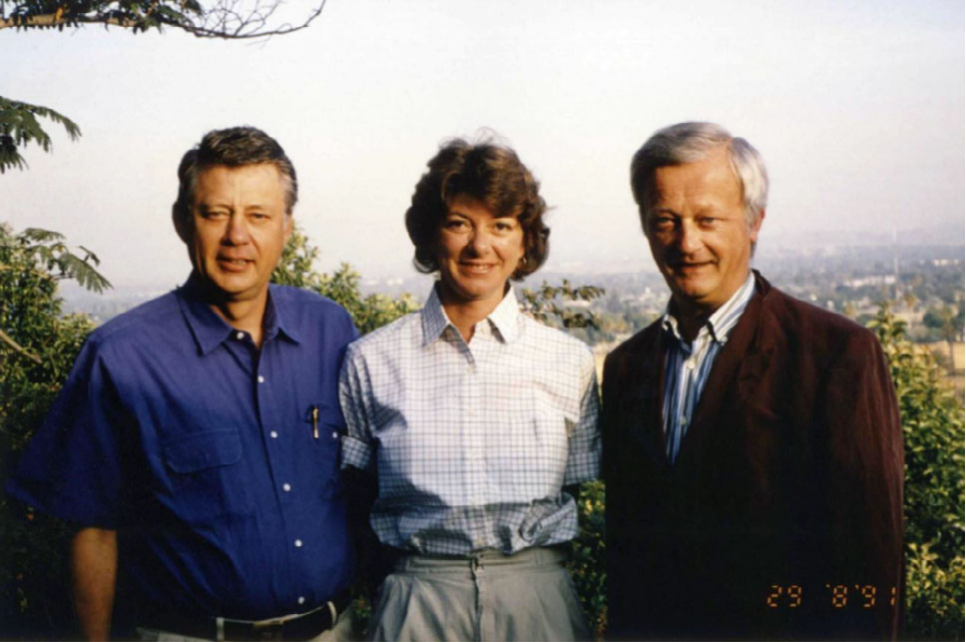
Anthony Norman, Helen Henry, and Roger Bouillon in Riverside, California, 1991.

**Fig 2:**
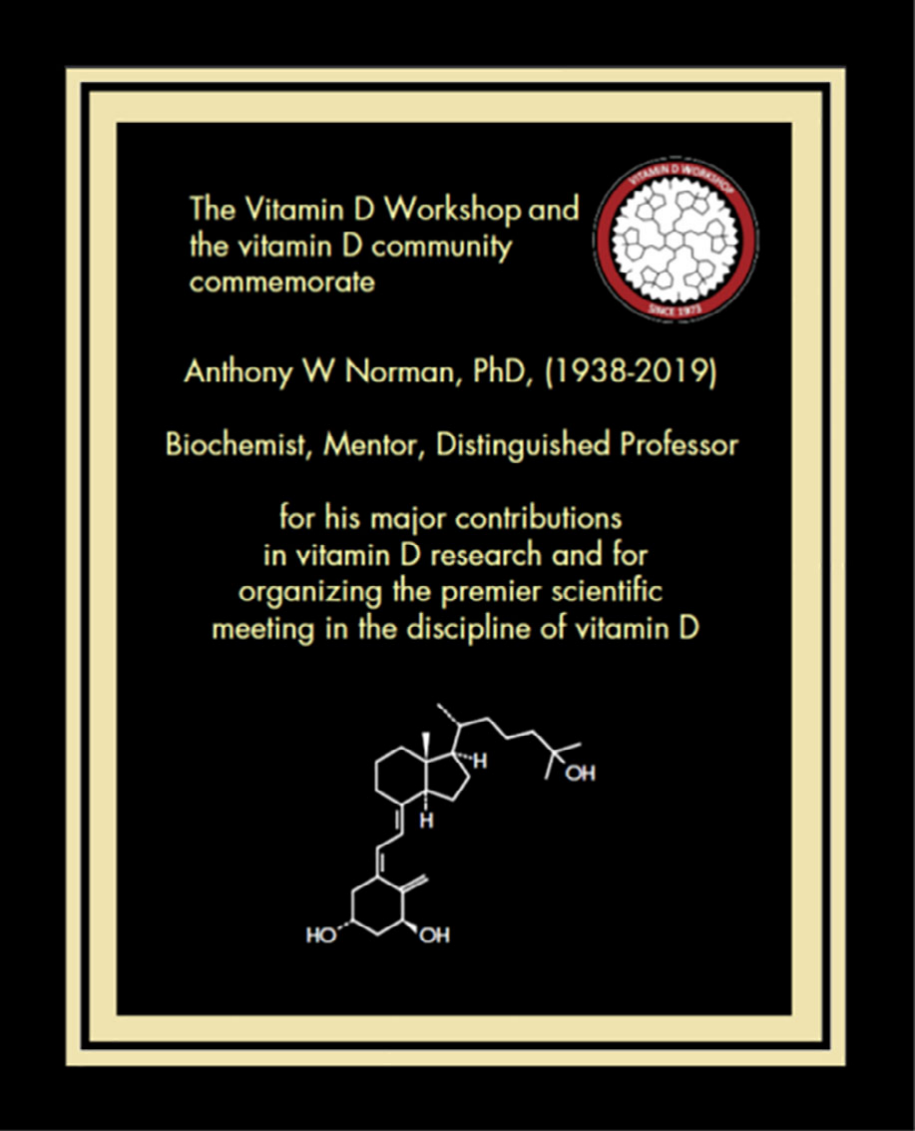
Plaque given to the Norman family on behalf of the Vitamin D Workshop at a commemorative service at the University of California, Riverside, September 2019.

Tony Norman was acutely aware of the importance of sharing scientific data with the whole world to promote the exchange of new information and ideas and to stimulate international collaboration. Therefore, he initiated a first small “workshop on vitamin D" in 1973 in Frankfurt (Germany), rapidly followed by a much larger meeting in Wiesbaden (Germany) in 1974. Thereafter, the vitamin D workshops were organized every 3 years, alternatively in North America and Europe. Because of the rapid expansion of vitamin D research, annual workshops were organized from 2012 onwards. The specific scientists responsible for the workshop organization varied over time,^2^, ^3^ but there is no doubt that Tony Norman was the leader, who took on the largest organizational burden of these meetings. Workshop attendance over time increased and exceeded 600 participants at the 8^th^ Workshop in Paris (1991). After the 15th annual meeting in Houston (2012), the organization of the workshops was transferred elegantly to a rotating workshop executive committee, ensuring an efficient and equitable system for the administration of future workshops. The 23rd annual meeting (2020) was projected to be organized for the first time outside of North America or Europe—on Australia's Gold Coast. Unfortunately, the COVID‐19 pandemic prohibited the assembly of vitamin D scientists, leading to the postponement of this meeting until 2022.

Tony Norman inspired the vitamin D community for nearly 50 years by the organization of the workshops and the publication of the proceedings of each meeting. Therefore, the vitamin D community owes it to him to commemorate this important aspect of his scientific career.

Mark Haussler, Dan Bikle, Sylvia Christakos, JoEllen Welsh, and Roger Bouillon thought that a special issue of *JBMR Plus* on many aspects of vitamin D might well be a long‐lasting legacy to commemorate Anthony W Norman in concert with the spirit of his ideas about promoting science via international workshops and publications. Fortunately, the Editor‐in‐Chief of *JBMR Plus* immediately agreed to assume the responsibility for editing and publishing this special issue, with editorial assistance from the above‐mentioned initiators of this volume. Approximately 30 vitamin D research groups were invited to submit a manuscript and, not surprisingly, there was overwhelming enthusiasm to participate in this heartfelt endeavor. Many manuscripts were submitted and entered the peer‐review process of the *Journal*; the first series of manuscripts accepted for publication is now ready for inclusion in the inaugural edition of the special issue of *JBMR Plus* to appear in January 2021. Additional accepted articles will be published in subsequent regular issues of *JBMR Plus*, designated as part of the tribute to AW Norman. Ultimately, all of the tribute manuscripts will be published, in toto, as a compendium that can be saved in honor of Tony's impact on the vitamin D field.

Please look forward to vitamin D from A to Z in *JBMR Plus* in 2021! The following topics will be at least touched upon and many covered in‐depth in a contemporary fashion: absorption of calcium, bone/brain, cancer, diabetes, epithelia, FGF23/food, genomic 1,25(OH)_2_D action, hydroxylases, immune system modulation/infection/inflammation/intestine, jejunum, kidney/Klotho, liver/lung, metabolism/muscle, nervous system/nutrition, ossification/osteoblast/osteocyte, pancreas/ PTH, quantification, receptor/reabsorption of phosphate/resorption, skin, T‐cell/transport, UV light, vitamin D history, Wnt‐signaling, X‐ray/X‐receptor, YES1 tyrosine protein kinase, and zinc fingers.

The deputy editor and associate editors are indebted not just to the contributing authors. They also thank Editor‐in‐Chief Peter Ebeling as well as Jessica Downey, who tirelessly and superbly supervised this project, and Wiley for its support of this special issue that will forever memorialize the legend of Tony Norman as the ultimate vitamin D aficionado.

## Author Contributions

**Roger Bouillon:** Conceptualization; writing‐original draft; writing‐review and editing. **Mark Haussler:** Conceptualization; writing‐review and editing. **Daniel Bikle:** Conceptualization; writing‐review and editing. **Sylvia Christakos:** Conceptualization; writing‐review and editing. **JoEllen Welsh:** Conceptualization; writing‐review and editing.

### Peer Review

The peer review history for this article is available at https://publons.com/publon/10.1002/jbm4.10445.
